# Lipid hijacking: A unifying theme in vector-borne diseases

**DOI:** 10.7554/eLife.61675

**Published:** 2020-10-29

**Authors:** Anya J O'Neal, L Rainer Butler, Agustin Rolandelli, Stacey D Gilk, Joao HF Pedra

**Affiliations:** 1 Department of Microbiology and Immunology, University of Maryland School of MedicineBaltimoreUnited States; 2 Department of Microbiology and Immunology, Indiana University School of MedicineIndianapolisUnited States; University of GenevaSwitzerland; University of GenevaSwitzerland

**Keywords:** vector-borne diseases, arthropod vectors, infection

## Abstract

Vector-borne illnesses comprise a significant portion of human maladies, representing 17% of global infections. Transmission of vector-borne pathogens to mammals primarily occurs by hematophagous arthropods. It is speculated that blood may provide a unique environment that aids in the replication and pathogenesis of these microbes. Lipids and their derivatives are one component enriched in blood and are essential for microbial survival. For instance, the malarial parasite *Plasmodium falciparum* and the Lyme disease spirochete *Borrelia burgdorferi*, among others, have been shown to scavenge and manipulate host lipids for structural support, metabolism, replication, immune evasion, and disease severity. In this *Review*, we will explore the importance of lipid hijacking for the growth and persistence of these microbes in both mammalian hosts and arthropod vectors.

## Shared resource utilization by diverse organisms

**Figure 1.**
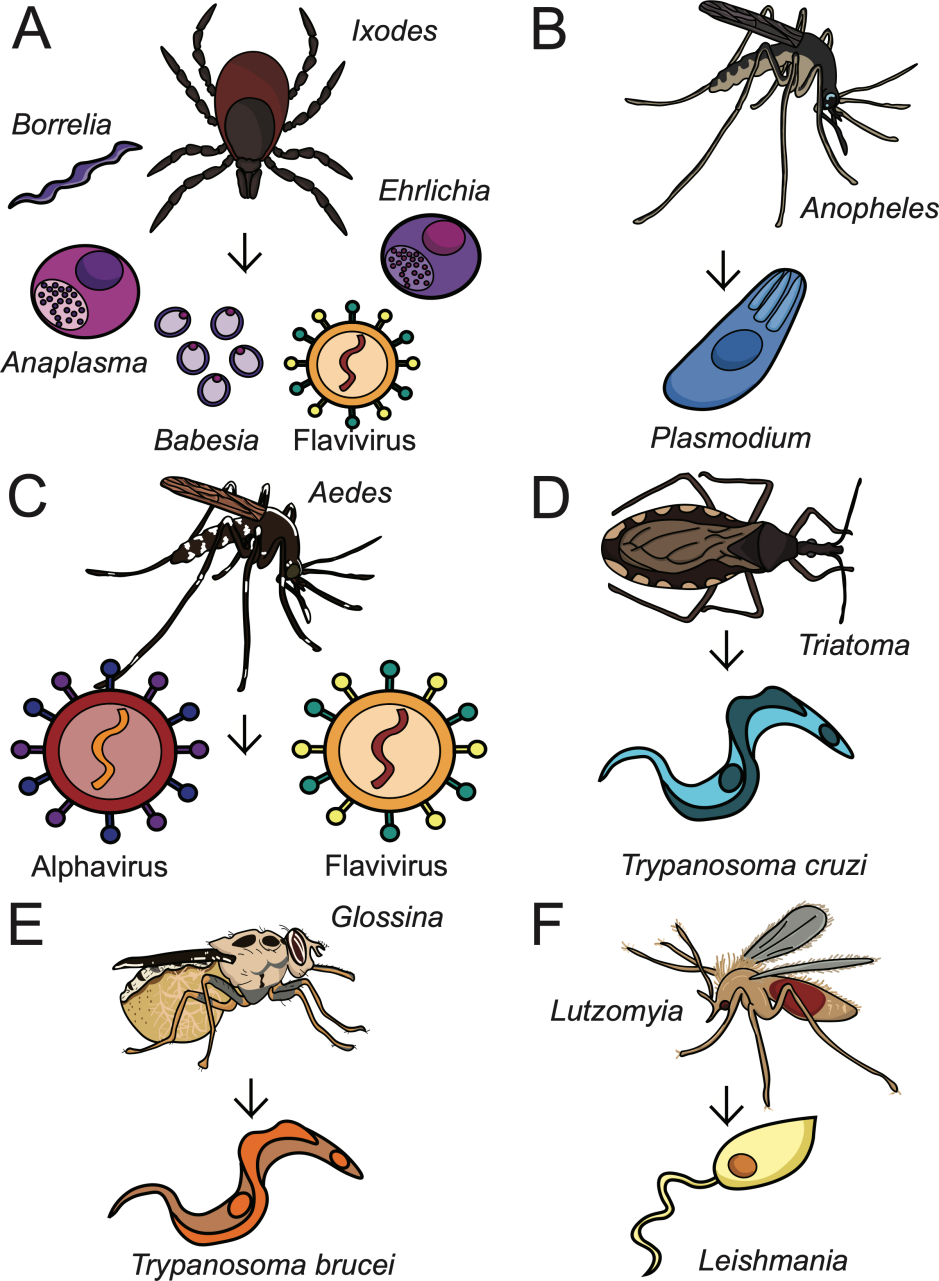
Arthropod-borne pathogens and their vectors. Arthropod vectors transmit various bacteria, viruses, and parasites to mammalian hosts. These pathogens infect hundreds of millions of people each year and are a primary concern of public health efforts (WHO, 2020). (**A**) *Ixodes* spp. ticks transmit POWV, *Borrelia*, *Anaplasma*, *Ehrlichia*, and *Babesia* spp.; (**B**) *Anopheles* mosquitoes transmit *Plasmodium* spp.; (**C**) *Aedes* (shown) and *Culex* mosquitoes transmit various flaviviruses and alphaviruses; (**D**) *Triatoma* (shown) and *Rhodnius* triatomines transmit *Trypanosoma cruzi*; (**E**) *Glossina* tsetse flies transmit *Trypanosoma brucei*; and (**F**) *Lutzomyia* (shown) and *Phlebotomus* sand flies transmit *Leishmania* spp.

Vector-borne diseases contribute to hundreds of millions of infections each year and are a primary focus of global public health efforts (WHO, 2020). These illnesses are caused by pathogens spread by blood feeding arthropods, or vectors, and include mosquitoes, ticks, sandflies, fleas and triatomines. During a blood meal, vectors may transmit an array of microbes depending on the arthropod species specificity and global pathogen distribution. For example, the tick genus *Ixodes* transmits bacterial, viral, and parasitic agents throughout the northern hemisphere (Eisen and Eisen, 2018; Figure 1A). *Anopheles* and *Aedes* mosquitoes can harbor malarial parasites and arboviruses, respectively, and are found on all continents excluding Antarctica (Neafsey et al., 2015; Leta et al., 2018; Figure 1B and C). Triatomines, tsetse flies and sand flies are responsible for the spread of trypanosomatid parasites in Latin America, Africa, and Asia (Vieira et al., 2018; Lukeš et al., 2018; Figure 1D–F). Despite evolving specific host-pathogen relationships, vector-borne microbes all share the requirement for blood nutrition during acquisition and transmission.

These pathogens spend a significant portion of time in the bloodstream and are surrounded by a variety of nutrients. Some of the compounds found abundantly in the blood are lipids, which include fatty acids and cholesterols. Lipids function as membrane components, signaling molecules and energy sources in prokaryotic and eukaryotic cells. Interestingly, decades of research reveal many vector-borne pathogens have a unique dependence on host lipids for survival. For instance, tick-borne bacteria, including *Borrelia*, *Anaplasma*, and *Ehrlichia* spp. are some of the few bacterial species known to accumulate host cholesterol in their membranes (Lin and Rikihisa, 2003a; Toledo and Benach, 2015). *Plasmodium* spp. require lipid building blocks from mammalian and mosquito hosts to complete their life cycles (Atella et al., 2009; Costa et al., 2018; Kilian et al., 2018). The life cycle of flaviviruses is intimately associated with host lipid metabolism and synthesis pathways (Leier et al., 2018). It is important to note that microbes cannot synthesize cholesterol, and many of these pathogens are limited in their ability to synthesize certain lipids or preferentially acquire lipids from the host (Fraser et al., 1997; Lin and Rikihisa, 2003a; Ramakrishnan et al., 2013).

Vector-borne pathogens have long fascinated scientists from multiple disciplines, including, but not limited to, microbiologists, immunologists, entomologists, epidemiologists, clinicians, and public health professionals. Recently, the scientific community has also become increasingly interested in the metabolic response to infection. The rising use of metabolomics has improved and expanded our understanding of biological processes, including lipid metabolism of vector-borne infections. However, a comprehensive and unifying *Review* Article describing the basic framework of lipid utilization by these pathogens is lacking.

In this *Review*, we aim to convey the shared role of host lipid hijacking in vector-borne diseases. We will explore how extracellular and intracellular microbes scavenge lipids from hosts, how they manipulate host factors and metabolism for invasion and growth and how they use lipids in immune evasion and disease severity. We will also describe mechanisms of lipid utilization in both mammalian hosts and arthropod vectors. Finally, we will discuss outstanding questions in the fields of microbiology and vector biology and how cutting-edge technologies may be applied. By defining shared mechanisms of resource exploitation by diverse pathogens, we hope to draw attention to the importance of these processes and how future studies could lead to novel strategies in the treatment or prevention of vector-borne diseases.

## Classification of lipids

**Figure 2.**
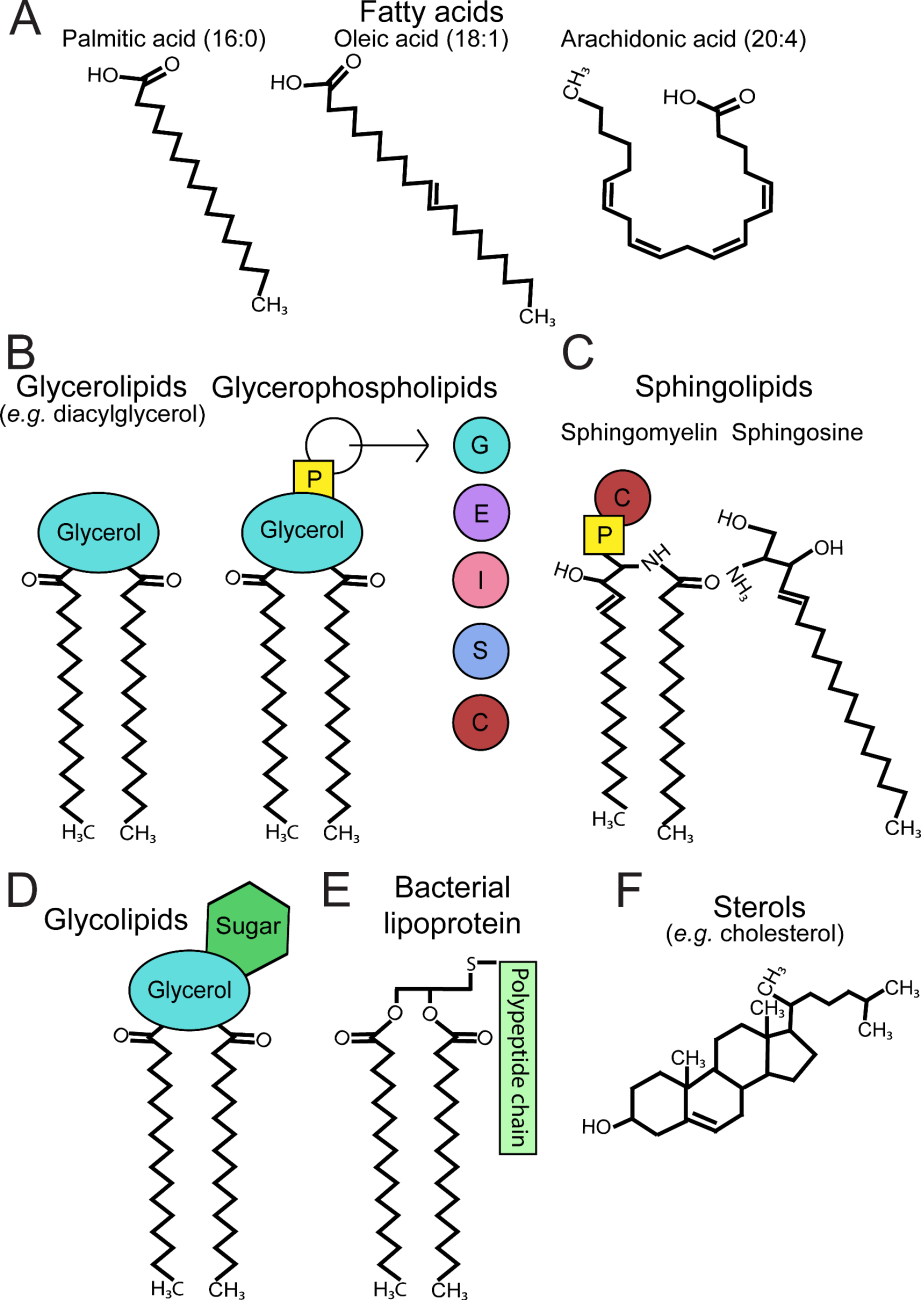
Classification of membrane lipids. Lipids structure the membranes of both eukaryotic and prokaryotic cells. (**A**) Fatty acids are hydrophobic building blocks for many membrane lipids. Common fatty acids include palmitic acid (16:0), oleic acid (18:1), and arachidonic acid (20:4), whose structures differ in the number of carbons and number of double bonds per chain. The presence of double bonds in the chains of certain fatty acids, such as oleic acid and arachidonic acid, makes these lipids unsaturated. (**B**) Glycerolipids and glycerophospholipids are two classifications of lipids found in eukaryotic and prokaryotic membranes. Glycerolipids (e.g. diacylglycerol) contain a glycerol backbone and at least one fatty acyl chain. Glycerophospholipids, conversely, are glycerolipids that contain a polar head group comprised of phosphate and an alcohol, and are named accordingly (e.g. PG, PE, PI, PS, PC). In the membrane, these lipids largely consist of two fatty acyl chains connected to the glycerol backbone. (**C**) Sphingolipids are membrane lipids that possess a sphingosine backbone. These include sphingomyelin, which contains a sphingosine backbone, one fatty acyl chain, and either phosphocholine (shown) or phosphoethanolamine. (**D**) Glycolipids refer to membrane lipids, such as glycerolipids or sphingolipids, that are covalently linked to a sugar residue. (**E**) Bacterial lipoproteins are membrane molecules that possess an exposed polypeptide chain and either two (diacyl; shown) or three (triacyl) fatty acyl chains. (**F**) Sterols are essential in eukaryotic physiology and include the animal sterol cholesterol, a critical component of cell membranes. p = phosphate, G = glycerol, E = ethanolamine, I = inositol, S = serine, C = choline, PG = phosphatidylglycerol, PE = phosphatidylethanolamine, PI = phosphatidylinositol, PS = phosphatidylserine, PC = phosphatidylcholine.

Lipids are hydrophobic compounds that structure cellular membranes, activate signaling pathways and provide energy to cells (Figure 2). They are defined by their insolubility in water and solubility in organic solvents (Fahy et al., 2011). There are eight main classifications of lipids: fatty acyls, glycerolipids, glycerophospholipids, sphingolipids, sterols, prenols, saccharolipids, and polyketides (Fahy et al., 2011). Fatty acyls include fatty acids (e.g. palmitate and oleate, both found abundantly in the human body) and make up the hydrophobic components of complex lipids (Figure 2A).

Glycerolipids and glycerophospholipids are two groups that comprise a significant portion of prokaryotic and eukaryotic membranes (Figure 2B). They both contain a glycerol backbone and at least one fatty acyl chain. However, they differ in their presence or absence of a polar head group (Figure 2B). Conversely, sphingolipids are membrane lipids that contain a sphingosine backbone. Sphingomyelin, a lipid found in animal cell membranes, is an example (Figure 2C). Generally speaking, bacteria and parasites are capable of synthesizing phospholipids and sphingolipids but may prefer scavenging available host lipids or animal-specific lipids.

Glycolipids consist of membrane lipids that are covalently linked to a sugar residue (Figure 2D). Glycolipids are not to be confused with saccharolipids, which have fatty acid chains directly linked to a sugar backbone. The most well-known example of saccharolipids is lipid A, a component of lipopolysaccharide found in Gram-negative bacterial membranes. Bacterial membranes may also contain lipoproteins on their surface, which possess both fatty acyl and polypeptide components (Figure 2E). Additionally, sterols are essential in eukaryotic physiology and include cholesterol, a component of animal cell membranes (Figure 2F). Other eukaryotes, such as parasites and fungi, may synthesize alternative sterols (e.g. ergosterol).

Importantly, lipids can act as signaling mediators. Lipid signaling molecules include sterols, lysophospholipids (a subclass of glycerophospholipids consisting of one fatty acyl chain and a polar head group), phosphoinositides (PIPns) and eicosanoids, which are derived from arachidonic acid (Figure 2A) or other polyunsaturated fatty acids. Lipid mediators, particularly eicosanoids, can be produced during infection and may promote or inhibit inflammation (Dennis and Norris, 2015). Phosphoinositides are derived from phosphatidylinositol (PI) and interact with intracellular proteins to generate second messengers, such as inositol trisphosphate (IP_3_) and diacylglycerol (DAG) (Falkenburger et al., 2010). Although PIPn signaling has been implicated as a critical process during pathogenesis, we will primarily refer to literature on eicosanoids and lysophospholipids regarding lipid signaling. An extensive classification of lipid structures is reviewed elsewhere (Fahy et al., 2011).

## Lipid scavenging by extracellular pathogens

**Figure 3.**
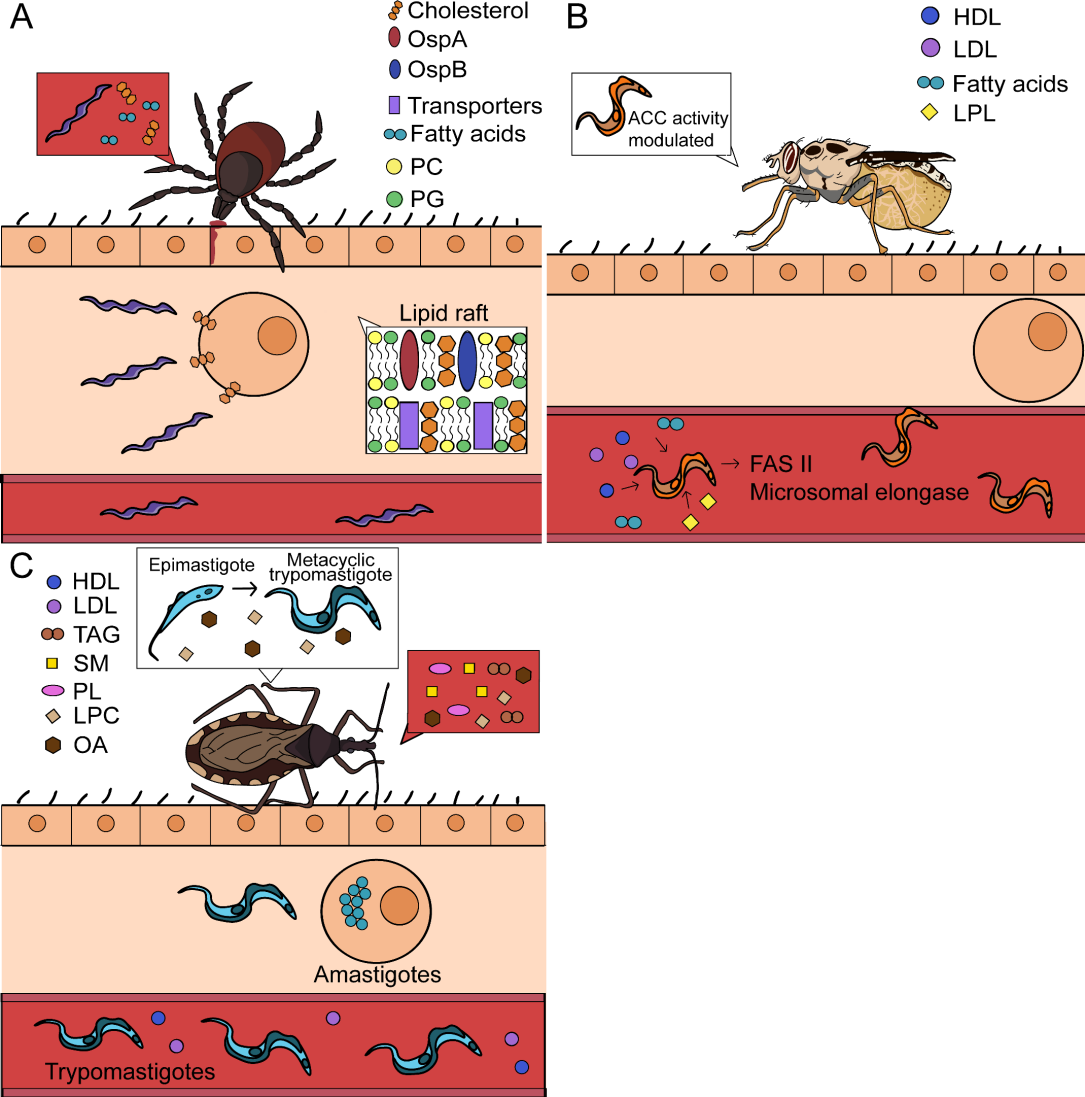
Lipid scavenging by extracellular arthropod-borne pathogens. Certain pathogens can obtain nutrients directly from their environment and may not require host cell invasion for lipid uptake. (**A**) *Borrelia* spp. are extracellular bacteria and acquire lipids directly from mammalian cells and the blood. *B. burgdorferi* also organizes its membrane into eukaryotic-like lipid rafts. (**B**) *Trypanosoma brucei* is extracellular in both mammalian and arthropod hosts. These parasites acquire various lipids from the blood and can synthesize their own fatty acids using the type II fatty acid synthase system (FASII) and the microsomal elongase pathway when resources are restricted. In the tsetse fly, the enzyme acetyl coenzyme A carboxylase (ACC) regulates the microsomal elongase pathway based on lipid abundance. (**C**) *Trypanosoma cruzi* is extracellular in its trypomastigote and epimastigote forms. Within the triatomine, epimastigotes acquire various lipids from the blood meal. Additional lipids function as signaling molecules and promote the differentiation from epimastigotes into metacyclic trypomastigotes. At this stage, metacyclic trypomastigotes may be deposited in the skin of a mammal before invading cells to become amastigotes. Intracellular amastigotes transform into trypomastigotes, burst out of host cells, and enter the bloodstream. PC = phosphatidylcholine, PG = phosphatidylglycerol, HDL = high density lipoprotein, LDL = low density lipoprotein, LPL = lysophospholipid, TAG = triacylglycerol, SM = sphingomyelin, PL = phospholipid, LPC = lysophosphatidylcholine, OA = oleic acid.

Membrane formation is a critical step in microbial proliferation. Microbes are generally divided into extracellular or intracellular organisms based on their metabolic requirements and lifecycles. Extracellular microbes often harbor their own replication and synthesis machinery. However, vector-borne pathogens may lack lipid synthesis enzymes and must acquire these components from the host (Figure 3). In this section, we will discuss how extracellular vector-borne pathogens scavenge host lipids for survival.

### Borrelia

*Borrelia* is a genus of tick-borne, extracellular spirochetes that includes *B. burgdorferi*, the causative agent of Lyme disease. They are loosely considered ‘Gram-negative’ bacteria; yet, their membranes lack characteristic Gram-negative features such as lipopolysaccharide (LPS) and diaminopimelic acid in their peptidoglycan layer (Yanagihara et al., 1984; Takayama et al., 1987). Instead, *Borrelia* spp. use phospholipids, cholesterols and lipoproteins for structural support of their bacterial envelope (Brandt et al., 1990; Belisle et al., 1994; Toledo and Benach, 2015). The incorporation of host lipids is strongly tied to their unique membrane formation. In this *Review,* we will refer to studies on Lyme disease-causing *Borrelia*, also known as the *B. burgdorferi* sensu lato complex.

*B. burgdorferi* is capable of phospholipid synthesis and predominantly incorporates phosphatidylglycerol (PG) and phosphatidylcholine (PC) into its membrane (Hossain et al., 2001; Wang et al., 2004). However, these bacteria lack the enzymes to synthesize fatty acids, such as palmitate or oleate (Fraser et al., 1997). Therefore, they acquire these fatty acids from hosts and integrate them into bacterial phospholipids and lipoproteins (Belisle et al., 1994; Hossain et al., 2001; Figure 3A). Lipoproteins are an immunogenic component of *Borrelia* membranes and have several functions, including critical roles in the colonization of the blacklegged tick *Ixodes scapularis* (Brandt et al., 1990; Pal et al., 2000; Pal et al., 2004). Despite their extracellular lifecycle, there is evidence that *B. burgdorferi* infection influences lipid metabolism in the host. A recent study in Lyme disease patients demonstrated that *B. burgdorferi* infection increases serum levels of lysophosphatidylcholine (LPC), a variant of PC that contains one fatty acyl chain (Fitzgerald et al., 2020). Lysophospholipids are generated as a byproduct of phospholipid degradation or an intermediate of phospholipid synthesis. Lysophospholipid accumulation during bacterial infection may be a result of bacterial stress, lipoprotein synthesis, or host response to infection (Zheng et al., 2017). However, the significance of this increase during Lyme disease has yet to be explored.

Bacteria cannot synthesize cholesterol. However, *Borrelia* are one of a few bacterial species that integrate host cholesterol into their membranes (Toledo and Benach, 2015; Figure 3A). In mammals, *B. burgdorferi* scavenges cholesterol directly from the plasma membranes of host cells (Crowley et al., 2013). It may also acquire cholesterol from the blood meal, as ticks fed on mice infected with *Borrelia* spp. display dysregulated cholesterol levels compared to uninfected controls (Hoxmeier et al., 2017). Uniquely, *B. burgdorferi* organizes cholesterol and cholesterol glycolipids into eukaryotic-like lipid rafts on their inner or outer membranes (LaRocca et al., 2010; Toledo et al., 2018). Inner and outer membrane lipid rafts appear to be distinct from each other. For example, outer membrane rafts contain well-defined lipoproteins involved in colonization, such as the outer membrane proteins (Osp) A and B, while inner membrane rafts possess proteins with signaling and transport functions, including ATP-binding cassette transporters (Toledo et al., 2014; Toledo et al., 2018). OspA and OspB were also shown to selectively associate with cholesterol glycolipids found in lipid rafts, while the lipoprotein OspC did not, suggesting the specificity of their interactions contributes to lipid raft composition (Toledo et al., 2014; Toledo et al., 2015b). Thus, these bacteria rely on host lipids for membrane organization, structure, and survival.

### Trypanosomatida

Trypanosomatids, which include *Trypanosoma cruzi*, *Trypanosoma brucei*, and *Leishmania* spp., are single-cell flagellated parasites that cause a high burden of disease in developing countries. *T. cruzi* and *T. brucei* are the etiological agents of Chagas disease and African sleeping sickness respectively, while *Leishmania* spp. cause leishmaniasis (Field et al., 2017). *T. cruzi* and *Leishmania* spp. are extracellular in the arthropod vector and transmitted to mammals in their trypomastigote (*T. cruzi*) or promastigote (*Leishmania*) forms. These parasites will then infect mammalian cells and undergo intracellular stages. Conversely, *T. brucei* is an exclusively extracellular parasite. Trypanosomatids have developed different strategies to survive under dynamic physiological conditions, including fluctuations in available lipids during their life cycles (Lukeš et al., 2018; Landfear and Zilberstein, 2019; Figure 3B and C).

Extracellular trypanosomatids scavenge host lipids to support their proliferation. In the mammalian host, trypanosomatids internalize low-density lipoprotein (LDL) and high-density lipoprotein (HDL), whose hydrolysis can yield fatty acids and sterols for parasite growth (Gillett and Owen, 1992; Coppens et al., 1995; Andrade-Neto et al., 2011; De Cicco et al., 2012). They may also acquire free lipids in the serum, including free fatty acids and lysophospholipids for their membranes (Voorheis, 1980; Berman et al., 1987; Bowes et al., 1993). However, if there is an exogenous limitation on fatty acids, *T. brucei* may synthesize these lipids de novo from ketogenic carbon sources (Millerioux et al., 2018; Figure 3B). *T. brucei* may endogenously produce fatty acids from the type II fatty acid synthase pathway (FASII) or from the microsomal elongase pathway, which uses the enzyme acetyl coenzyme A carboxylase (ACC) (Millerioux et al., 2018; Ray et al., 2018). The activity of this enzyme is regulated by lipid abundance in the tsetse fly, therefore maintaining a balance between lipid scavenging and synthesis (Ray et al., 2018).

In the triatomine vector, *T. cruzi* epimastigotes use lipids from the blood meal for differentiation and membrane composition (Figure 3C). The blood meal contains components such as phospholipids, triacylglycerols, and sphingomyelin that are quickly degraded into free fatty acids by the vector. These lipids are then incorporated into epimastigotes (Wainszelbaum et al., 2003). Epimastigotes may scavenge additional lipid building blocks from the host to synthesize their surface molecules. For example, *T. cruzi* requires inositol for many of their membrane components, such as glycoinositolphospholipids, and utilizes transporters to acquire it for their synthesis (Einicker-Lamas et al., 2007). Interestingly, the blood meal possesses signaling lipids, including oleic acid, LPC, and lysophosphatidic acid that promote the differentiation and proliferation of these parasites in triatomines (Wainszelbaum et al., 2003; Chagas-Lima et al., 2019). If epimastigotes internalize excess lipids, they store them in specialized lipid organelles for later use (Pereira et al., 2018). Ultimately, these extracellular pathogens rely on host lipids to complete their life cycles.

## Cellular invasion

There are a variety of strategies intracellular microbes employ to successfully invade host cells. Often, host lipids such as membrane cholesterol are co-opted for cellular entry. Cholesterol regulates host cell plasma membrane fluidity and helps structure lipid rafts (Simons and Ehehalt, 2002). For example, the *Rickettsiales* are a group of obligate intracellular bacteria and comprise the tick-borne bacterial genera *Anaplasma* and *Ehrlichia. A. phagocytophilum* and *E. chaffeensis*, two human pathogens transmitted by Ixodidae ticks, infect granulocytes and monocytes, respectively (McClure et al., 2017). To invade cells, these bacteria utilize cholesterol-containing lipid rafts in host membranes for entry (Lin and Rikihisa, 2003b). Lipid rafts often contain receptors required for entry and enable a cascade of signaling events leading to engulfment of the pathogen by the host cell (Simons and Ehehalt, 2002).

Membrane cholesterol is also important for arboviral invasion. Alphaviruses are the only genus of the *Togaviridae* family and are positive sense, single-stranded RNA mosquito-borne viruses, including Chikungunya (CHIKV), Semliki Forest (SFV), and Sindbis (SINV) viruses. They have broad tropism and can cause severe pathology by infecting neurons in the central nervous system (Atkins, 2013). Alphaviruses enter cells through the endocytic pathway, where the acidic pH of the endosomes causes the fusion of viral and endosome membranes and allows viral RNA to be released into the cytoplasm (Phalen and Kielian, 1991). Membrane cholesterol was found to play a critical role in the ability of SFV and SINV to fuse with the endosomal membrane, as viral fusion and replication were interrupted in cholesterol-depleted cells (Phalen and Kielian, 1991; Lu et al., 1999). Interestingly, neither SFV nor SINV rely on lipid rafts to successfully fuse with target membranes, suggesting they require cholesterol for membrane fluidity (Waarts et al., 2002).

Intracellular parasites may use membrane lipids and host membrane repair pathways to facilitate entry. Invading *Leishmania* and *T. cruzi* require membrane cholesterol similar to other intracellular pathogens (Pucadyil et al., 2004; Fernandes et al., 2007; Kumar et al., 2016). However, unlike the alphaviruses, *T. cruzi* utilizes lipid rafts containing sphingolipids, cholesterol and the ganglioside GM1 for cellular entry (Fernandes et al., 2007; Barrias et al., 2007; Hissa et al., 2012). The mechanism of *T. cruzi* invasion is unique because it triggers a plasma membrane repair pathway to drive internalization (Tardieux et al., 1992). After induction of a membrane lesion or injury, lysosomes are recruited to the host plasma membrane to provide membranes for repair. These lysosomes contain acid sphingomyelinase (ASM), an enzyme that converts sphingomyelin to ceramide, which promotes the endocytosis of membrane lesions. It was shown that *T. cruzi* creates lesions in the host cell plasma membrane and uses recruited lysosomal membranes to create the parasitophorous vacuole, or replication niche (Tardieux et al., 1992; Fernandes et al., 2011). These parasites then require ASM to generate ceramide and drive internalization of the parasite by endocytosis (Fernandes et al., 2011). Thus, *T. cruzi* co-opts lipid membrane repair processes to successfully invade host cells.

Some parasites and viruses enter cells through host lipid receptors and transporters. *Flaviviridae* are a family of positive sense, single-stranded RNA viruses with diverse tropisms, including dengue (DENV), yellow fever (YFV), West Nile (WNV), Zika (ZIKV), and Powassan (POWV) viruses. Mosquitoes transmit the majority of these pathogens, excluding POWV, which is a member of the tick-borne encephalitis virus (TBEV) complex. Flaviviral entry has been shown to be impacted by interactions with lipid-binding proteins. DENV infection is enhanced by the interaction between viral particles and serum apolipoprotein A-I, which is a major component of HDL (Li et al., 2013). This interaction assists DENV entry through scavenger receptor class B type I (SR-BI), a surface lipid transporter, and leads to severe disease (Li et al., 2013). Similarly, the parasite *T. cruzi* exploits other lipid receptors for invasion by binding serum lipoproteins. *T. cruzi* binds LDL, which may interact with LDL receptor (LDLR) to facilitate fusion and entry into the cell (Nagajyothi et al., 2011).

Additional lipid receptors, including phosphatidylserine (PS) receptors, play important roles in arboviral invasion. PS receptors are phospholipid-binding proteins that mediate the removal of apoptotic cells by phagocytosis. Two of these receptor families, TIM and TAM, were shown to facilitate DENV entry by interacting with PS on viral envelopes (Meertens et al., 2012). TIM1 appears to promote the entry and replication of other enveloped arboviruses as well, including various alphaviruses (Jemielity et al., 2013). Taken together, these examples emphasize that vector-borne microbes utilize membrane lipids and lipid receptors to provide an avenue for entry.

## Intracellular growth

Intracellular pathogens require resources and machinery to survive in host cells. These microbes often have reduced genomes through evolutionary selection and cannot synthesize necessary products on their own, including lipids required for membrane formation. Thus, pathogens hijack host enzymes and biosynthesis pathways to promote replication and growth (Table 1). The dual host environment requires vector-borne pathogens to rewire both arthropod and mammalian metabolism. How intracellular arthropod-borne infections utilize host lipids to promote growth is discussed below.

**Table 1.** Lipid hijacking by intracellular arthropod-borne pathogens.

Microbe	*Anaplasma and Ehrlichia*	Flaviviruses	Alphaviruses
Vector	• Accumulation of lipid transport and absorption proteins from the blood meal in the vector (Villar et al., 2016)	• Increase phospholipid and sphingolipid synthesis (Perera et al., 2012; Melo et al., 2016; Chotiwan et al., 2018; Vial et al., 2019) • Replication occur on ER; form replication vesicles but not convoluted membranes (Junjhon et al., 2014) • Increase aminophospholipid concentrations by regulating AGPAT1 expression in mosquito cells (DENV) (Vial et al., 2019) • Maintain cholesterol levels in mosquito cells by downregulating LRP-1 (DENV) (Tree et al., 2019) • Increase lipid droplets in the cell (Barletta et al., 2016)	• Increase lipid droplets in the cell (Barletta et al., 2016)
Mammal	• Accumulate cholesterol in membranes, elevate cellular cholesterol levels and traffic cholesterol to inclusions using flotillins and NPC1-bearing vesicles (*A. phagocytophilum*) (Xiong et al., 2009; Xiong and Rikihisa, 2012; Xiong et al., 2019) • Manipulate glycerolipid synthesis (*A. phagocytophilum*) (POPG, PODAG, MPPC) (Shaw et al., 2017) • Recruit membrane phospholipids to bacterial vacuoles (*E. chaffeensis*) (Lin et al., 2020)	• Viral replication curves the ER lipid bilayer into vesicles and convoluted membranes (Leier et al., 2018) • Increase sphingolipid synthesis and require ceramide for replication vesicles (WNV, ZIKV) (Martín-Acebes et al., 2014; Leier et al., 2020) • Recruit host fatty acid synthase to replication sites for fatty acid synthesis (DENV) (Heaton et al., 2010) • Increase intracellular cholesterol levels (DENV, WNV), induce cholesterol accumulation at the ER (DENV, WNV), and bring in HMGCR to replication sites for cholesterol synthesis (DENV) (Mackenzie et al., 2007; Rothwell et al., 2009; Soto-Acosta et al., 2013; Soto-Acosta et al., 2017) • Interact with lipid droplets on the ER for assembly (DENV) (Samsa et al., 2009) • Lipid droplets are consumed/reabsorbed by the ER for fatty acids and energy (DENV) (Heaton and Randall, 2010; Peña and Harris, 2012; Zhang et al., 2018)	• Require intracellular cholesterol transport (CHIKV) (Wichit et al., 2017) • Nonstructural proteins colocalize with lipid droplets (Remenyi et al., 2017) • Use Akt pathway to drive fatty acid synthesis (SFV) (Mazzon et al., 2018)
Microbe	*Plasmodium*	*Babesia*	
Vector	Oocysts: • Acquires lipids through lipophorin (Atella et al., 2009; Costa et al., 2018) • Availability of lipophorin-transported lipids impacts growth in the mosquito and infectivity, virulence, quantity, and metabolism of sporozoites (Costa et al., 2018; Werling et al., 2019)	Unknown	
Mammal	Liver stage: • Scavenges PC and extracellular and intracellularly synthesized cholesterol (Labaied et al., 2011; Itoe et al., 2014) • Promotes host lipid biosynthesis by inhibiting AMPK pathway (Kluck et al., 2019) • Uses parasite fatty acid synthesis (FAS) II pathway in late liver stage (Yu et al., 2008; Vaughan et al., 2009) Blood stage: • Scavenges fatty acids, choline, ethanolamine, and serine from blood to synthesize phospholipids (PC, PE) (Mikkelsen et al., 1988; Wein et al., 2018; Tanaka et al., 2019) • Uses host Kennedy and PMT pathways to synthesize phospholipids (Ancelin and Vial, 1989; Pessi et al., 2004) • Upregulates triacylglycerol, diacylglycerol, PG, etc. (Gulati et al., 2015) Sexual stages: • PMT pathway is required for making gametocytes (Bobenchik et al., 2013) • Availability of polyunsaturated fatty acids and downregulation of lysophospholipids in the blood triggers gametocytogenesis (Brancucci et al., 2017; Tanaka et al., 2019) • Gametocytes have decreased phospholipids, increased ceramides and sphingolipids (Gulati et al., 2015)	• Causes low HDL levels in patients (Cunha et al., 2000; Bock et al., 2017) • Causes low HDL levels in canines and cattle (Goodger et al., 1990; Milanović et al., 2019) • Synthesizes PC in the blood stage (Florin-Christensen et al., 2000)	
Microbe	Leishmania*	Trypanosoma cruzi*	
Mammal	• Increases lipid concentrations by regulating expression of lipid metabolism genes (Osorio y Fortéa et al., 2009; Rabhi et al., 2012; Semini et al., 2017) • Sequesters cholesterol in parasitophorous vacuoles and incorporates it in membranes; retains cholesterol using host V-ATPases (Tewary et al., 2006; Semini et al., 2017; Pessoa et al., 2019) • Induces lipid body formation (Rabhi et al., 2016) • Scavenges phospholipids and sphingolipids to make parasite-specific lipids, *e.g.* the sphingolipid inositol phosphorylceramide (Winter et al., 1994; Henriques et al., 2003; Zhang et al., 2005) • Contains increased cholesterol and free fatty acids, decreased ergosterol and triglycerides (Bouazizi-Ben Messaoud et al., 2017)	• Scavenges long chain fatty acids from pools of triacylglycerols for membranes (Gazos-Lopes et al., 2017) • Upregulates levels of cholesterol and LDL (Johndrow et al., 2014) • Requires host fatty acid oxidation for parasite growth (Caradonna et al., 2013; Li et al., 2016) • Induces lipid body formation (D'Avila et al., 2011)	

*Extracellular stages in the vector.

### Bacteria

The intracellular tick-borne bacteria *A. phagocytophilum* and *E. chaffeensis* interact with host lipids for replication. Following invasion, infectious bacteria, termed dense-core cells, establish a niche within host vacuoles. Then, these bacteria convert into a non-infectious, replicative form (reticulate cells) and undergo rapid division. Intracellular bacteria must traffic host resources and components, including lipids, to their replicative niche (Table 1). The replicating bacteria form morulae in the cell, convert back into their infectious dense-core form, and eventually are released by exocytosis or lysis (McClure et al., 2017).

Similar to *Borrelia, Anaplasma* and *Ehrlichia* spp. do not have LPS or diaminopimelic acid and instead utilize cholesterol and other lipids for their membranes (Lin and Rikihisa, 2003a). Thus, they have developed ways to hijack host lipid pathways to complete replication. Cholesterol accumulation and exploitation mechanisms have been well defined in these agents (Samanta et al., 2017). *A. phagocytophilum* obtains its cholesterol from exogenous LDL and will increase LDLR expression to elevate total cellular cholesterol (Xiong et al., 2009). Following the hydrolysis of LDL, *A. phagocytophilum* hijacks cholesterol trafficking pathways in the cell. For example, *A. phagocytophilum* targets flotillins and Neimann-Pick type C1 (NPC1)-bearing vesicles to redirect cholesterol to bacterial inclusions (Xiong and Rikihisa, 2012; Xiong et al., 2019). It was also shown that inhibiting cholesterol efflux from lysosomes halts *A. phagocytophilum* infection, further demonstrating their dependence on host cholesterol (Cockburn et al., 2019).

Moreover, these bacteria manipulate host glycerolipid synthesis for proliferation (Shaw et al., 2017; Lin et al., 2020). *E. chaffeensis* depends on host glycerolipids and uses autophagy and endocytosis to traffic host membrane lipids to vacuoles (Lin et al., 2020). Interestingly, the recruitment of glycerophospholipids leads to vesicle formation within bacterial inclusions, potentially acting as a membrane reserve for quickly replicating *E. chaffeensis* (Lin et al., 2020). Furthermore, these bacteria may prefer specific glycerolipids for their proliferation. During infection with *A. phagocytophilum*, these bacteria increase levels of 1-palmitoyl-2-oleoyl-sn-glycero-3-phosphoglycerol (POPG), 1-palmitoyl-2-oleoyl-sn-glycero-3-diacylglycerol (PODAG) and 1-myristoyl-2-palmitoyl-sn-glycero-3-phosphocholine (MPPC) in human HL60 cells (Shaw et al., 2017). Although the source of these lipids has not been identified, POPG and PODAG were demonstrated to activate immune pathways in the tick, suggesting these lipids are molecular patterns of infection (Shaw et al., 2017).

The role of lipid metabolism in the vector, *Ixodes scapularis*, is less understood. Lipid transport and absorption proteins from the blood meal may accumulate in tick midguts infected with *A. phagocytophilum* implicating a role for lipid utilization in the arthropod (Villar et al., 2016). To date, changes in tick lipid metabolism have only been observed during fungal and *B. burgdorferi* infection (Hoxmeier et al., 2017; Sá et al., 2018). Whether mechanisms of lipid utilization by intracellular bacteria are conserved during tick colonization remains to be studied.

### Viruses

The lifecycle of many flaviviruses are intimately tied to host lipids and aid in their replication and assembly (Leier et al., 2018; Table 1). Following cellular entry, these viruses uncoat and release their genome into the cytoplasm. The genome is translated into a polyprotein and cleaved into components that mediate replication. These viral proteins will then curve mammalian ER lipid bilayers into unique subcellular structures such as convoluted membranes and vesicles where viral replication occurs (Welsch et al., 2009; Gillespie et al., 2010; Cortese et al., 2017). Assembled virions bud off from the ER and undergo additional steps in the Golgi.

Because flaviviruses cannot make lipids on their own, they alter the cellular lipidome to promote replication. For example, flaviviruses manipulate host cholesterol to accomplish a number of functions including entry, replication, assembly, and budding (Osuna-Ramos et al., 2018). DENV and WNV upregulate intracellular cholesterol levels, induce its accumulation at the ER and require it for the formation of replication sites (Mackenzie et al., 2007; Rothwell et al., 2009; Soto-Acosta et al., 2013; Soto-Acosta et al., 2017). Because cholesterol is important for membrane remodeling, it is likely that these viruses use host cholesterol to maintain the membrane architecture of replication complexes (Soto-Acosta et al., 2017).

Another important component of flavivirus replication complexes are sphingolipids. The sphingolipid ceramide is required for vesicle biogenesis, an important process during flaviviral replication, and may be generated by the degradation of sphingomyelin (Trajkovic et al., 2008). It was shown that both WNV and ZIKV increase cellular sphingolipid levels, and blocking the conversion from sphingomyelin to ceramide inhibits their replication (Martín-Acebes et al., 2014; Martín-Acebes et al., 2016; Leier et al., 2020). Using ZIKV, it was also demonstrated that ceramide is redistributed to replication membranes, suggesting their involvement in flaviviral vesicle formation (Leier et al., 2020). Patients infected with flaviviruses exhibit lipidomic changes, such as dysregulated sphingolipids levels and perturbed lipid metabolic pathways, pointing to a critical process during viral infection (Cui et al., 2013; Melo et al., 2017).

How these viruses acquire host lipids is heavily studied. During the formation of ER invaginations, host factors are recruited to replication complexes to provide lipids for structure and energy. For instance, DENV recruits host enzymes, such as fatty acid synthase and the cholesterol synthesis enzyme 3-hydroxy-3-methyl-glutaryl-CoA reductase (HMGCR), to synthesize lipids at replication sites (Heaton et al., 2010; Soto-Acosta et al., 2013; Soto-Acosta et al., 2017). Specifically, DENV uses HMGCR to elevate cholesterol and increases its activity through impaired AMPK phosphorylation (Soto-Acosta et al., 2017). Flaviviruses also interact with host lipid droplets (LD), which are ER-derived organelles that store lipids for energy reserves and immunity. DENV increases the numbers of LDs in the cell and promotes their consumption by autophagy (Samsa et al., 2009; Heaton and Randall, 2010; Peña and Harris, 2012). They trigger LD consumption by interacting with the LD protein AUP1 to drive virus production (Zhang et al., 2018). Furthermore, these viruses use LDs for viral assembly. DENV capsid proteins colocalize to LDs, and their availability controls viral particle formation (Samsa et al., 2009). Collectively, these viruses have evolved a multitude of processes for acquiring necessary lipids from the mammalian host.

While not as abundantly studied as the flaviviruses, the alphaviruses display some reliance on host lipids. Similar to DENV, CHIKV requires intracellular cholesterol transport for replication and its nonstructural proteins localize to LDs, although the significance of this interaction is unclear (Wichit et al., 2017; Remenyi et al., 2017). During SINV infection, mice defective in ASM accumulate intracellular lipids that promote a greater production of infectious particles (Ng et al., 2008). Because ASM is known to regulate cholesterol and sphingolipid levels, these may be implicated in their proliferation. Alphaviruses may also shift the metabolism of the cell to make lipids. It was shown that SFV uses the Akt pathway to drive glucose metabolism towards fatty acid synthesis (Mazzon et al., 2018). Thus, there may be uncovered mechanisms of lipid utilization by these arboviruses.

An emerging field of arbovirus research explores their interactions with the mosquito vector. Insects are cholesterol auxotrophs; therefore, mosquito-borne viruses must co-opt different processes to obtain lipids in the vector. To maintain intracellular cholesterol, DENV downregulates expression of the low-density lipoprotein receptor-related protein 1 (LRP-1), a protein involved in cholesterol export that facilitates viral replication in mosquitoes (Tree et al., 2019). Interestingly, while the induction of DENV replication vesicles is conserved between mammals and mosquitoes, infected mosquito cells do not display convoluted ER membranes, possibly due to the lack of cholesterol synthesis (Junjhon et al., 2014). Instead, flaviviruses alter the lipidome of *Aedes* mosquitoes by increasing phospholipid and sphingolipid synthesis, likely for their replication and membrane formation (Perera et al., 2012; Melo et al., 2016; Chotiwan et al., 2018; Vial et al., 2019). DENV accomplishes this by regulating expression of acylglycerolphosphate acyltransferase (AGPAT1), which increases phospholipid concentrations and promotes infectivity (Vial et al., 2019). Finally, the interactions between arboviruses and LDs appear to be conserved, as both flaviviruses and alphaviruses induce their formation in mosquitoes (Barletta et al., 2016). Mosquito LD biogenesis was shown to be a direct result of immune activation; however, whether these viruses require mosquito LDs is unclear (Barletta et al., 2016).

### Parasites

#### Apicomplexa

The Apicomplexa are a phylum of intracellular protozoans. These parasites are named after the presence of an apicoplast, a unique organelle that aids in parasite metabolism (Vaughan et al., 2009). Two families of vector-borne apicomplexans are *Plasmodium* and *Babesia* spp., which are transmitted by mosquitoes and ticks, respectively.

*Plasmodium* spp. are the causative agents of malaria and undergo various stages in the human host. Mosquitoes deposit sporozoites in the skin, which migrate to the liver to infect hepatocytes and later erythrocytes in the bloodstream. The liver is a central regulator of lipid and fatty acid metabolism. During the liver stage, parasites require membrane phospholipids to undergo rapid replication (Table 1). *Plasmodium* scavenge PC directly from hepatocytes despite encoding enzymes for the type II fatty acid synthesis (FAS II) pathway and other lipid enzymes (Déchamps et al., 2010; Itoe et al., 2014). The prokaryotic FAS II pathway localizes to the apicoplast and appears to be involved in late liver stage development of *Plasmodium* spp. (Yu et al., 2008; Vaughan et al., 2009). Liver stage parasites also acquire host lipids by scavenging internalized and de novo synthesized cholesterol in hepatocytes, as well as promoting host lipid biosynthesis through the inhibition of the AMPK pathway (Labaied et al., 2011; Kluck et al., 2019).

Merozoites from the liver then progress to the bloodstream. During the blood stage, parasites synthesize their own phospholipids, as erythrocytes are unable to synthesize lipids de novo (Kilian et al., 2018; Table 1). *Plasmodium* acquire lipid building blocks, such as fatty acids, choline, ethanolamine, and serine from the serum and hemoglobin of infected individuals (Mikkelsen et al., 1988; Wein et al., 2018; Tanaka et al., 2019). These parasites then use the Kennedy and phosphatidylethanolamine methyltransferase (PMT) pathways of the host to generate phosphatidylethanolamine (PE) and PC, two of the predominant phospholipids found in blood-stage parasites (Ancelin and Vial, 1989; Pessi et al., 2004). Interestingly, the parasite FAS II pathway does not appear to contribute to blood stage development (Yu et al., 2008; Vaughan et al., 2009).

Host lipid metabolism is tightly regulated during both the asexual and sexual blood stages. Lipidomic studies reveal an upregulation of lipids such triacylglycerol, diacylglycerol and PG in *P. falciparum*-infected erythrocytes (Gulati et al., 2015). The shift from asexual to sexual blood stages is mediated by several factors, including the PMT pathway and regulation of lysophospholipid and fatty acid levels (Bobenchik et al., 2013; Gulati et al., 2015; Brancucci et al., 2017; Tanaka et al., 2019). For example, the host downregulates serum LPC levels as a physiological response to infection, which triggers *Plasmodium* gametocytogenesis (Brancucci et al., 2017). The lipid composition of gametocytes also differs from other stages, including increased ceramides and sphingolipids, suggesting a shift in lipid metabolism during sexual differentiation (Gulati et al., 2015).

Following the sexual stages, *Plasmodium* gametocytes are taken up by *Anopheles* mosquitoes and develop into oocysts. Proliferating oocysts acquire mosquito lipids through the uptake of the major lipid transporter lipophorin (Atella et al., 2009; Costa et al., 2018; Table 1). Recent studies have demonstrated the importance of lipophorin to parasite development, metabolic activity, and transmission. One study showed that the availability of lipophorin-transported lipids in the mosquito impacts parasite infectivity and virulence in the mammalian host (Costa et al., 2018). Furthermore, the restriction of these lipids affects the quantity and metabolic activity of transmitted sporozoites (Costa et al., 2018). A separate study revealed that steroid hormone signaling by 20-hydroxyecdysone (20E) positively regulates development of mosquito eggs and *Plasmodium* oocysts (Werling et al., 2019). When 20E signaling is disrupted, oocysts utilize lipids transported by lipophorin to accelerate parasite growth and become infectious sooner, remarkably without affecting the fitness of the mosquito (Werling et al., 2019). Given that 20E and other lipophorins have reported roles in antiplasmodial immunity, these studies exhibit an intricate relationship between lipid utilization by *Plasmodium* and vector competence (Gupta et al., 2010; Reynolds et al., 2020).

*Babesia* parasites infect several mammalian hosts and replicate in erythrocytes. Compared to *Plasmodium*, there is less known about *Babesia* pathogenesis. However, patients infected with *Babesia* display lipid abnormalities and markedly low HDL levels, suggesting these parasites depend on host lipid metabolism as well (Cunha et al., 2000; Bock et al., 2017; Table 1). Similar abnormalities have been observed in *Babesia*-infected cattle and canines (Goodger et al., 1990; Milanović et al., 2019). These parasites have been demonstrated to rewire lipid biosynthesis to produce PC during the blood stage, much like *Plasmodium* (Florin-Christensen et al., 2000). Similar to other tick-borne infections, their interactions with lipids in the tick remain unknown.

#### Trypanosomatida

*T. cruzi* and *Leishmania* spp. have intracellular stages in the mammalian host. Following transmission by its vector, *Leishmania* promastigotes infect macrophages and differentiate into obligate intracellular amastigotes within phagolysosomes (Loría-Cervera and Andrade-Narvaez, 2020). *T. cruzi* trypomastigotes also infect mammalian cells and develop into intracellular amastigotes; conversely, they replicate predominantly in the cytosol and can infect a variety of tissues, including muscle and adipose tissue (Lewis and Kelly, 2016). Trypanosomatids may synthesize many of their necessary lipids de novo, including fatty acids, sphingolipids, and phospholipids (Zhang and Beverley, 2010). However, their intracellular forms require lipid salvaging mechanisms (Table 1).

Trypanosomatids contain significant amounts of inositol phosphorylceramide (IPC), which is a sphingolipid common in fungi (Zhang and Beverley, 2010; Zhang et al., 2010). In *Leishmania* spp., the lipid rafts of these parasites are enriched in IPC and ergosterol, a substitute for mammalian cholesterol found in fungi and protozoa (Zhang and Beverley, 2010). Although promastigotes can synthesize IPC, differentiated amastigotes lose this ability but retain significant amounts of this lipid (Zhang et al., 2005). Studies demonstrate that after transitioning from promastigotes to amastigotes, *Leishmania* likely scavenge and remodel host lipids for parasite-specific molecules such as IPC (Winter et al., 1994; Henriques et al., 2003; Zhang et al., 2005). *Leishmania* amastigotes also exhibit changes in lipid composition compared to promastigotes, such as decreased levels of triglycerides and ergosterol and increased levels of fatty acids and cholesterol, suggesting a shift toward mammalian lipid scavenging (Bouazizi-Ben Messaoud et al., 2017).

Much like other intracellular pathogens, trypanosomatids obtain necessary lipids through the rewiring of host pathways. *Leishmania* and *T. cruzi* control intracellular lipid concentrations by elevating intracellular cholesterol and regulating expression of lipid metabolism genes (Osorio y Fortéa et al., 2009; Rabhi et al., 2012; Johndrow et al., 2014; Semini et al., 2017). *Leishmania* sequesters exogenous cholesterol in parasitophorous vacuoles before incorporating it into their own membranes (Tewary et al., 2006; Semini et al., 2017). To retain sequestered cholesterol, they route host V-ATPases to parasitophorous vacuoles and maintain cholesterol homeostasis (Pessoa et al., 2019). Additionally, although not well understood, both *Leishmania* and *T. cruzi* induce the formation of LDs in infected cells, suggesting a role in energy consumption (D'Avila et al., 2011; Rabhi et al., 2016).

For *T. cruzi*, the ability to balance de novo lipid synthesis with host lipid scavenging is an active area of research. These parasites produce their own fatty acids and sterols and were shown to upregulate enzymes involved in their synthesis during intracellular infection (Li et al., 2016). Regardless, intracellular growth of *T. cruzi* is tied to host lipids. Through a genome-wide RNAi screen, genes involved in host fatty acid oxidation were reported as being critical for the growth of *T. cruzi* amastigotes, thus linking host metabolism to parasite replication (Caradonna et al., 2013). Furthermore, amastigotes were shown to utilize long chain fatty acids from pools of host triacylglycerols in order to fuel their proliferation (Gazos-Lopes et al., 2017). Taken together, intracellular vector-borne pathogens draw on several different mechanisms to accomplish similar lipid scavenging tasks.

## Immune evasion and disease severity

**Figure 4.**
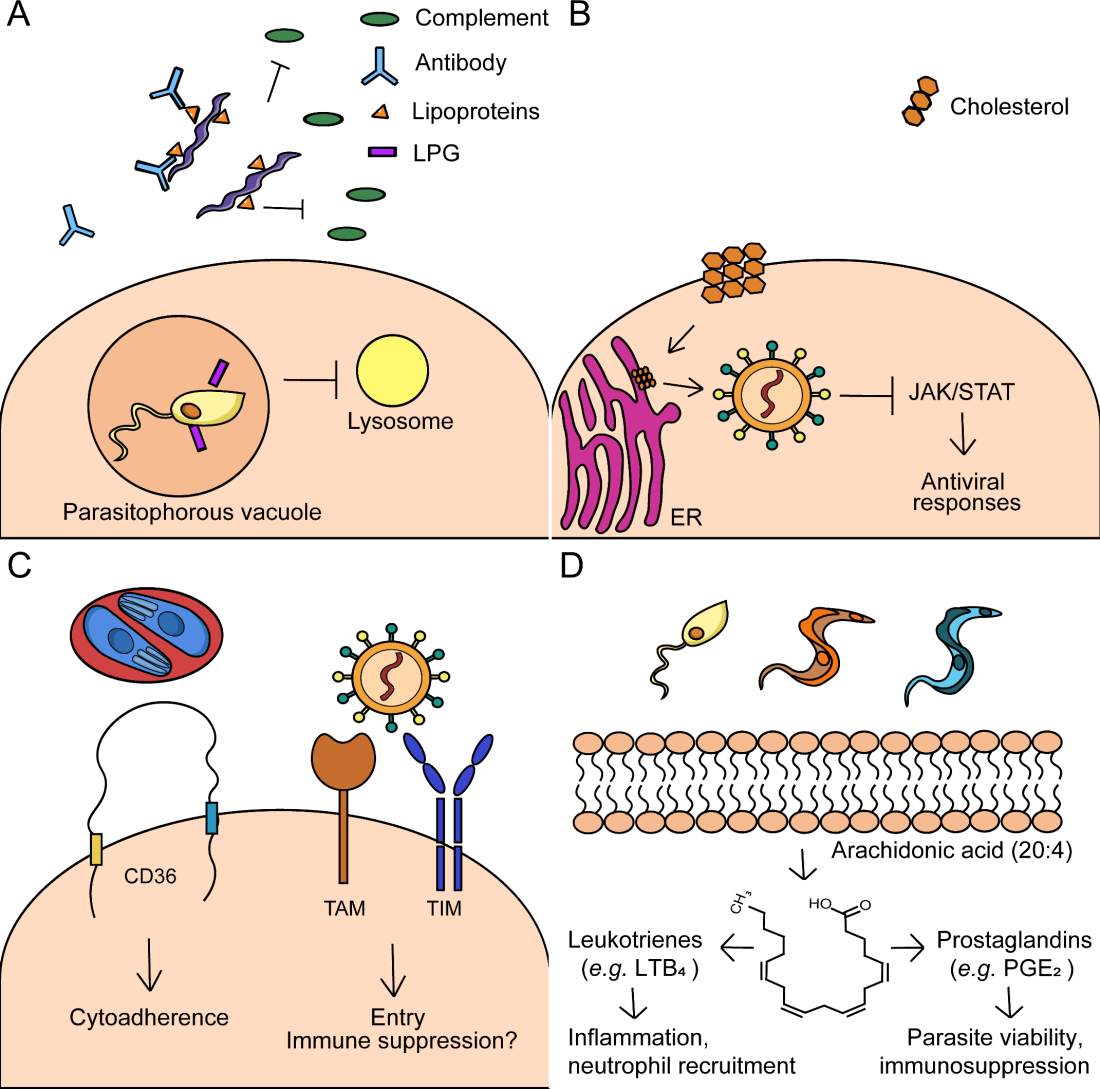
Immune evasion mechanisms by arthropod-borne pathogens. (**A**) *Microbial lipids*: *Borrelia* spp. use lipoproteins and other surface lipids to evade immunity and promote disease in humans. *Borrelia* possess various lipoproteins that inhibit the complement system in the blood. Additionally, antibodies are generated during *Borrelia* infection that promote anti-phospholipid syndrome, an autoimmune condition that targets phospholipids. *Leishmania* spp. contain the surface molecule lipophosphoglycan (LPG), which prevents the maturation of the phagosome. (**B**) *Host lipids and metabolism:* West Nile Virus (WNV) redistributes cholesterol from the plasma membrane to replication sites at the ER. This phenomenon disrupts lipid rafts, which downregulates JAK/STAT activation and antiviral responses. (**C**) *Surface lipid receptors:* Erythrocytes infected with *Plasmodium* engage the lipid receptor CD36. Infected erythrocytes bind CD36 on endothelial cells and promote cytoadherence, which may lead to severe complications (e.g. cerebral malaria). Flaviviruses and arboviruses have been shown to engage the phosphatidylserine (PS) receptors TIM and TAM. These receptors have known immunosuppressive functions. Engagement of TAM, specifically, has been shown to inhibit type I interferon signaling during infection with other enveloped viruses. (**D**) *Lipid signaling molecules:* Eicosanoids, such as prostaglandins and leukotrienes, are formed by the cleavage of arachidonic acid. Eicosanoids may have immunomodulatory roles during trypanosomatid infections. Prostaglandin E_2_ (PGE_2_), for example, promotes parasite viability and may be immunosuppressive. Conversely, leukotriene B_4_ (LTB_4_) is proinflammatory and recruits neutrophils.

The mammalian and arthropod immune systems have evolved mechanisms to recognize and eliminate pathogens based on their microbial life cycles. Microbial survival and persistence in the host requires evasion of the immune system. Vector-transmitted pathogens have developed ways to subvert immunity through membrane lipids, host lipid metabolism, surface receptors, and lipid signaling (Figure 4). We will discuss how lipids help microbes evade host immune responses and how this contributes to disease severity.

### Bacteria

The reliance of host lipids by arthropod-borne bacteria is both critical to pathogen survival and intertwined with evasion mechanisms. *Borrelia* spp. possess an arsenal of lipoproteins that aid in mammalian persistence (Christodoulides et al., 2017). *Borrelia* lipoproteins are immunogenic and must be tightly regulated by environmental cues, including temperature, CO_2_ levels and immune pressure (Brandt et al., 1990; Schwan et al., 1995; Liang et al., 2004; Hyde et al., 2007; Wilder et al., 2016). *Borrelia* lipoproteins are known to undergo antigenic variation in mammalian hosts, specifically the variable major protein (VMP)-like sequence (Vls) E (Zhang et al., 1997; Zhang and Norris, 1998). This variation in VlsE is critical for reinfection with *Borrelia* and error-prone repair mechanisms during recombination contribute to this process (Rogovskyy and Bankhead, 2013; Verhey et al., 2018).

*Borrelia* lipoproteins can directly influence immune recognition and activation. Various surface lipoproteins derived from the *B. burgdorferi* sensu lato complex, including OspC, OspE, CRASP-1, CRASP-2, and BBK32 promote pathogen survival by binding or inactivating components of the complement pathways (Hellwage et al., 2001; Kraiczy et al., 2001; von Lackum et al., 2005; Garcia et al., 2016; Caine et al., 2017; Figure 4A). Similar mechanisms, such as the inactivation of factor H (FH) and FH-like protein 1 by CRASP-1, have been recently elucidated in *Borrelia mayonii*, an emerging Lyme disease agent (Walter et al., 2019). Although many of the complement evasion strategies identified in *Borrelia* are in vitro phenomena, the ability to inhibit complement may be critical for arthropod acquisition. For example, CRASP-1 mutants, which are unable to inhibit factor H, do not survive during blood meal and are unable to be transmitted to naive hosts (Hart et al., 2018). Furthermore, lipoproteins may display immunomodulatory effects against immune cells. OspB and OspC have been shown to inhibit phagocytosis by neutrophils and macrophages, respectively (Hartiala et al., 2008; Carrasco et al., 2015). OspA and OspB also have roles in modulating neutrophil oxidative bursts (Hartiala et al., 2008).

In addition to evasion, the relationship between tick-borne bacteria and host lipids can contribute to severe disease in humans. During an immune response, antibodies are produced to neutralize the microbes. The generation of antibodies to *Borrelia* lipids may contribute to severe pathologies during Lyme disease (Figure 4A). It was demonstrated that a significant portion of Lyme disease patients possess autoantibodies to apolipoprotein B-100 or annexin 2, which is a common determinant in the autoimmune disorder anti-phospholipid syndrome (Crowley et al., 2015; Pianta et al., 2015). Additionally, severe outcomes during Lyme disease may be associated with host metabolism. Patients with early disseminated Lyme disease have more pronounced metabolic signatures than those with early localized Lyme disease (Fitzgerald et al., 2020). One such metabolic signature is the differential production of eicosanoids that correlate with symptoms such as Lyme arthritis (Blaho et al., 2009; Fitzgerald et al., 2020). The shared requirement of cholesterol by *Borrelia* and *Anaplasma* spp. may influence their pathogenicity, as hypercholesterolemia and high-cholesterol diet were shown to promote *Borrelia* and *Anaplasma* infection, respectively (Xiong et al., 2007; Toledo et al., 2015a). Therefore, it can be speculated that additional shared mechanisms involving host lipids may lead to severe outcomes in disease during tick-borne infections.

### Viruses

The innate immune system promotes viral elimination through interferon signaling. Because arboviruses can expertly manipulate lipid metabolism, this can be used to thwart immunity. For example, the mammalian JAK/STAT pathway is activated by interferon and regulates antiviral responses and lipid metabolism (Xu et al., 2013). During WNV infection, cholesterol is redistributed from the plasma membrane to viral replication sites. This redistribution was demonstrated to disrupt lipid rafts and diminish JAK/STAT activation, which could then be restored by the addition of exogenous cholesterol (Mackenzie et al., 2007; Figure 4B). In the vector, the JAK/STAT pathway may also regulate lipid metabolism. Mosquitoes with constitutively active JAK/STAT signaling display resistance to DENV and downregulated expression of lipid metabolism genes, such as those involved in cholesterol transport (Jupatanakul et al., 2017). Therefore, it is conceivable that flaviviruses may use lipids to counteract these immune pathways in the mosquito. The interplay between cellular lipids and immune activation may also contribute to disease severity, as low LDL levels are associated with severe outcomes in patients infected with DENV (Biswas et al., 2015).

Arboviruses may mitigate immune responses prior to viral entry. The PS receptors TIM and TAM mediate both the removal of apoptotic cells and immunosuppressive responses (Szondy et al., 2017). Flaviviruses and alphaviruses have been shown to utilize TIM and TAM receptors for viral entry and TIM receptors for replication (Meertens et al., 2012; Jemielity et al., 2013). Notably, engagement of TAM receptors with enveloped viruses was shown to dampen innate immune responses by inhibiting type I interferon signaling (Bhattacharyya et al., 2013; Figure 4C). Thus, it is plausible that PS on flavivirus and alphavirus envelopes contributes to innate immune inactivation and evasion.

### Parasites

#### Apicomplexa

*Plasmodium* spp. primarily use protein antigens to evade host immunity. However, these parasites utilize host lipid receptors to invade other tissues and promote disease severity. One of the most defined mechanisms of host-*Plasmodium* interaction is with CD36, an evolutionarily conserved lipid scavenger receptor and transporter (Hsieh et al., 2016). CD36 contributes to apoptotic cell clearance, pathogen recognition and uptake of host lipids, including the potentially harmful form of cholesterol, oxidized LDL (oxLDL) (Febbraio et al., 2001). During *Plasmodium* infection, oxLDL is responsible for the phagocytosis of infected red blood cells (iRBCs), regulation of inflammatory responses and induction of cellular immunity by phagocytic immune cells (Serghides and Kain, 2001; Gowda et al., 2013; Thylur et al., 2017). However, CD36 has been implicated as a determinant of severe malarial infection (Figure 4C). Endothelial CD36 binds domains of the *Plasmodium falciparum* erythrocyte membrane protein 1 (PfEMP1) with high affinity, tethering iRBCs to the endothelium in a phenomenon called cytoadherence (Barnwell et al., 1989; Hsieh et al., 2016). Sequestration of iRBCs to the endothelium promotes parasite survival, potentially by preventing splenic clearance, and is tied to severe pathologies such as cerebral malaria (Fonager et al., 2012). In the mosquito vector, the CD36 ortholog Croquemort may also be involved in parasite recognition although this relationship is not explicitly defined (González-Lázaro et al., 2009).

In terms of disease severity, lipidomics has been used to distinguish clinical manifestations of malaria, including acute and chronic disease (Cordy et al., 2019). Susceptibility to severe malaria is correlated with decreased levels of eicosanoids, such as prostaglandin E_2_ (PGE_2_) in both *P. falciparum*- and *P. vivax*-infected patients (Perkins et al., 2001; Abreu-Filho et al., 2019). Studies from *P. vivax-*infected patients also reveal that parasitemia is significantly associated with metabolites involved in lipid metabolism, an observation that may be tied to their intracellular growth (Gardinassi et al., 2017). Conversely, in a study observing factors associated with pre-existing *P. vivax* immunity, semi-immune subjects exhibit increased levels of linoleate metabolites compared to naive subjects (Gardinassi et al., 2018). The ability of an infected individual to respond to malaria treatment may also be tied to host lipids, where patients with lower levels of baseline glycerophosphocholines were more likely to develop chloroquine-resistant malaria after infection (Uppal et al., 2017). Together, the relationship between clinical presentation and host lipidomics allows for the use of lipid biomarkers in patients with malaria.

#### Trypanosomatida

Trypanosomatids use membrane components and parasite lipids to evade the immune response. *Leishmania* promastigotes contain lipophosphoglycan (LPG), a critical virulence factor and lipid-containing polysaccharide (Späth et al., 2003). LPG has been well characterized for its role in inhibiting phagosome maturation and establishing infection in macrophages (Desjardins and Descoteaux, 1997; Moradin and Descoteaux, 2012; Figure 4A). In the vector, LPG facilitates arthropod colonization. This membrane component is critical for midgut attachment and protection from hydrolytic enzymes in the sand fly (Sacks et al., 2000; Kamhawi et al., 2004; Kamhawi, 2006; Myskova et al., 2007; Jecna et al., 2013). Other lipids have been implicated in *Leishmania* immune evasion as well. For example, infective parasites express higher levels of polyunsaturated fatty acid metabolites and may promote the differentiation of macrophages into a less inflammatory phenotype, known as the M2 phenotype (Paloque et al., 2019). These M2 macrophages produce high levels of pro-resolving bioactive lipids to facilitate parasite survival and proliferation (Paloque et al., 2019). For *T. cruzi*, these parasites possess membrane lipids that may be involved in the induction of severe disease. It was demonstrated that *T. cruzi* glycolipids activate a specific γδ-T cell population associated with cardiomyopathy, a severe outcome of Chagas disease (Passos et al., 2017).

Within the host cell, regulation of host cholesterol is critical for intracellular trypanosomatid survival. Paradoxically, cholesterol appears to have a protective effect against *Leishmania* and *T. cruzi* infections. *Leishmania* survival outside of the parasitophorous vacuole depends on lower cholesterol. For example, mice and patients infected with *Leishmania* exhibit decreased levels of serum cholesterol, which may be a result of downregulated microRNA expression by the parasite (Lal et al., 2007; Ghosh et al., 2013). This dependence on lower cholesterol extends to the plasma membrane, where reduced membrane cholesterol disrupts membrane lipid rafts and inhibits antigen presentation to T cells (Chakraborty et al., 2005). Additional studies have shown that hypocholesterolemia promotes host susceptibility to *Leishmania,* while the converse protects against infection (Ghosh et al., 2012; Ghosh et al., 2013; Ghosh et al., 2014). Similarly, cholesterol may be somewhat protective against severe *T. cruzi* infection, as mice fed a high fat diet had increased survival and less myocardial damage despite increased cardiac cholesterol levels (Nagajyothi et al., 2014). This was attributed to the ‘obesity paradox’, where obesity has been observed as having a positive effect on certain chronic disease outcomes (Nagajyothi et al., 2014).

Another shared mode of evasion and disease severity by these parasites is the differential expression of eicosanoids. Specifically, prostaglandins appear to promote immunosuppression and pathogenesis (Figure 4D). During *Leishmania* infection, PGE_2_ and prostaglandin F_2_α (PGF_2_α) were shown to be upregulated and enhance parasite viability (Araújo-Santos et al., 2014; França-Costa et al., 2015; Alves-Ferreira et al., 2020). PGE_2_ has an immunosuppressive effect during *T. cruzi* infection and can be derived from host or parasite LDs (Toledo et al., 2016; Lovo-Martins et al., 2018; de Almeida et al., 2018). Although less understood, this eicosanoid may be somewhat involved in *T. brucei* immunosuppression as well. *T. brucei* infection induces the generation of suppressor macrophages that inhibit T cell proliferation, a hallmark of African trypanosomiasis (Schleifer and Mansfield, 1993). The suppressive activities of these macrophages are partially mediated by PGE_2_, where it was shown that the inhibition of NO and prostaglandin synthesis together abrogates T cell suppression (Schleifer and Mansfield, 1993). Along with the host, *T. brucei* may produce PGE_2_ and PGF_2_α, although their functional significance is unclear (Kubata et al., 2000). Furthermore, PGE_2_ may contribute to clinical complications. The prostaglandin synthesis enzyme COX-2 and PGE_2_ signaling were shown to be important for cardiac inflammation during Chagas disease (Guerrero et al., 2015). The role of this eicosanoid in cardiomyopathy is controversial, however, as conflicting reports show that PGE_2_ may also be cardioprotective (Sharma et al., 2013).

The differential production of eicosanoids directly affects the capacity of the host to control trypanosomatid infection. Leishmaniasis manifests in several different types, including the milder presentation of localized cutaneous leishmaniasis and the more severe mucocutaneous leishmaniasis. Patients with mucocutaneous leishmaniasis display higher levels of leukotriene B_4_ (LTB_4_), a proinflammatory eicosanoid that recruits neutrophils, and decreased PGE_2_ compared to patients with localized cutaneous leishmaniasis, suggesting this enhanced inflammatory profile contributes to severe *Leishmania* (França-Costa et al., 2016). Whether LTB_4_ is produced for the benefit of the parasite or host is debatable. LTB_4_ has been shown to be critical for parasite killing by neutrophils and macrophages (Morato et al., 2014; Tavares et al., 2014). However, as *Leishmania* may infect neutrophils before being ingested by macrophages, it has been suggested that these parasites use LTB_4_ to recruit additional cells to the site of infection. *L. major* induces LTB_4_ during early neutrophil infection in a manner similar to *A. phagocytophilum*, which has a tropism for neutrophils (Plagge and Laskay, 2017). Thus, this could be a mechanism to promote early persistence of the pathogen.

In the insect vector, *Trypanosoma* have adopted additional strategies for evasion and transmission. *Trypanosoma rangeli*, another species native to Central and South America, shares many characteristics with *T. cruzi* but infects the insect salivary glands for transmission. To accomplish this, *T. rangeli* uses its surface glycolipids to subvert nitric oxide (NO) production in the salivary glands and persist in the vector (Gazos-Lopes et al., 2012). *Trypanosoma* parasites may also use lipids to promote successful transmission. *T. cruzi* parasites bind lipoproteins from the vertebrate blood meal on their surface, thus agglutinating parasites and enhancing infectivity in the insect vector (Moreira et al., 2018). These parasites may benefit from the immunosuppressive properties of arthropod-derived lipids, where LPC in the salivary glands of *Triatoma infestans* and *Rhodnius prolixus* was shown to enhance *T. cruzi* transmission by altering the local inflammatory environment at the bite site (Mesquita et al., 2008; Lima et al., 2018). Ultimately, the interactions between parasites, hosts, and lipids are critical for the establishment and dissemination of arthropod-borne infections.

## Conclusion and perspective

Recent advances in the field of metabolism have allowed for a deeper understanding of host-pathogen interactions. Because of their increasing global health significance, research about vector-borne pathogens has experienced a surging interest from the scientific community. Great strides have been made in investigating otherwise neglected tropical diseases, including *Trypanosoma* and *Leishmania*. Additional work in understudied microbes, such as *Babesia*, alphaviruses, and POWV, among others, is imperative for understanding shared mechanisms used by vector-borne pathogens. Using these microbes to study the molecular basis of infection could unlock new paradigms in microbiology.

The regulation of lipids is a crucial process in both mammalian and arthropod metabolism, and arthropod-borne pathogens have evolved mechanisms to co-opt these processes. For example, only seven bacterial genera are known to incorporate mammalian cholesterol in their membranes, including the tick-borne *Borrelia*, *Anaplasma*, and *Ehrlichia* (Toledo and Benach, 2015). Based on their shared environment, it is easy to speculate why tick-borne bacteria are overrepresented for this quality. Other discoveries have prompted the development of novel therapeutics for vector-borne diseases. For instance, both extracellular and intracellular arthropod-borne pathogens must salvage lipids from their hosts for survival (Table 2). To this end, drugs targeting lipid metabolism have been shown to inhibit arboviral and parasitic infections (Tewary et al., 2006; Bobenchik et al., 2013; Merino-Ramos et al., 2016; Choi et al., 2016; Jiménez de Oya et al., 2019). Thus, their use in humans is a promising area of research. Studying host lipid requirements by these pathogens can also aid in understanding host susceptibilities to infection, such as comorbidities and medications taken for other diseases.

**Table 2.** Requirement of lipid scavenging for microbial growth.

	Cholesterol	Fatty acids	Phospholipids	Sphingolipids
*Anaplasma and Ehrlichia*	Essential (Lin and Rikihisa, 2003a; Xiong et al., 2009; Cockburn et al., 2019)	Essential (Dunning Hotopp et al., 2006)	Essential (Lin et al., 2020)	Unknown
*Borrelia*	Essential (LaRocca et al., 2010; Crowley et al., 2013; Toledo et al., 2015a)	Essential (Belisle et al., 1994; Fraser et al., 1997; Hossain et al., 2001)	Non-essential (Hossain et al., 2001; Wang et al., 2004)	Unknown
Flaviruses	Essential (Rothwell et al., 2009; Soto-Acosta et al., 2013)	Essential (Heaton et al., 2010)	Essential (Vial et al., 2019)	Essential (Martín-Acebes et al., 2014; Leier et al., 2020)
*Plasmodium*	Unclear (Labaied et al., 2011)	Essential (mammal: blood) (Yu et al., 2008; Vaughan et al., 2009)	Essential (mammal: liver) (Déchamps et al., 2010; Itoe et al., 2014)	Non-essential (Zhang et al., 2010)
*Leishmania*	Essential (mammal) (Andrade-Neto et al., 2011; De Cicco et al., 2012; Rabhi et al., 2012; Semini et al., 2017)	Unknown	Essential (mammal) (Zhang and Beverley, 2010)	Essential (mammal) (Winter et al., 1994; Henriques et al., 2003; Zhang et al., 2005; Zhang and Beverley, 2010)
*T. brucei*	Essential (mammal) (Coppens et al., 1995)	Non-essential (Millerioux et al., 2018; Ray et al., 2018)	Non-essential (Ramakrishnan et al., 2013)	Non-essential (Zhang et al., 2010)
*T. cruzi*	Essential (mammal) (Johndrow et al., 2014)	Essential (mammal and vector) (Wainszelbaum et al., 2003; Caradonna et al., 2013; Li et al., 2016; Gazos-Lopes et al., 2017)	Non-essential (vector) (Ramakrishnan et al., 2013; Chagas-Lima et al., 2019)	Non-essential (Zhang et al., 2010)

The interactions between microbes and their arthropod vectors have also come to the forefront of vector biology research. Molecular strategies of vector competence, or the ability of an arthropod vector to efficiently transmit disease, are currently being defined in insect and non-insect arthropods. These include: (1) pathogen recognition by diverse arthropod immune systems, (2) secretion of salivary effector proteins, (3) survival advantages provided by colonized microbes, and (4) interactions with mammalian immunity and the microbiome at the bite site. This emerging area of research is critical for understanding why arthropod vectors are tolerant of certain human pathogens and how these interactions developed on an evolutionary scale.

Vector-borne microbes are required to adapt to the mammalian host and the arthropod environment. However, most microbes considered ‘human pathogens’ are not detrimental to the arthropod, which is a phenomenon that is not well understood. For example, lipid utilization by microbes does not appear to impair arthropod fitness, yet lipid dysregulation is often a symptom of severe disease in mammals (Ghosh et al., 2012; Biswas et al., 2015; Toledo et al., 2015a; Costa et al., 2018; Werling et al., 2019; Cordy et al., 2019; Fitzgerald et al., 2020). This observation is intriguing, considering the general lipid compositions of invertebrate vectors and mammalian hosts are fairly similar. They both store excess lipids in the form of triacylglycerols and intracellular lipid droplets, they synthesize fatty acids and phospholipids for cellular membranes, and hematophagous arthropods acquire mammalian lipids (e.g. cholesterol) during blood meal and incorporate them in their cellular processes (Arrese and Soulages, 2010; Tree et al., 2019). Although many of these processes are conserved across species, the role of metabolism in dual host adaptation remains to be defined. Perhaps, the metabolic requirements for the microbe differ in each environment or the arthropod vector supplies necessary metabolites that prevent pathogenesis. During DENV infection, for instance, mosquito cells produce a significant number of unidentified metabolites that may represent unannotated, mosquito-specific molecules (Chotiwan et al., 2018). A high-resolution comparison of human and arthropod metabolites under infection conditions, as well as a larger focus on understudied vectors (e.g. sand flies, tsetse flies, triatomines) may help answer these questions.

The emergence of new and systems-based technologies offers an exciting outlook for the fields of vector biology and microbiology. For example, metabolomics is a high-throughput technique that provides a full picture of cellular perturbations underlying disease processes. By studying levels of metabolites, researchers can interrogate thousands of molecular changes including those involved in lipid metabolism. Lipidomics has already been applied for several vector-borne pathogens and has been instrumental in understanding host-pathogen interactions (Blaho et al., 2009; Perera et al., 2012; Cui et al., 2013; Gulati et al., 2015; Gardinassi et al., 2017; Gazos-Lopes et al., 2017; Melo et al., 2016; Melo et al., 2017; Chotiwan et al., 2018; Cordy et al., 2019; Vial et al., 2019; Leier et al., 2020); however, its further implementation with hypothesis-driven research may uncover universal mechanisms shared by several microbes. Additionally, the rise of cutting-edge technologies, such as single-cell metabolomics and mass spectrometry imaging, allows researchers to take a systems biology approach to answering metabolic questions. Mass spectrometry imaging reveals spatial distribution of individual metabolites, such as lipids, which can be applied to individual tissues from mammals or arthropods. Single-cell metabolomics can provide insight into the heterogeneity of infection and how individual cells respond to infection. Thus, a more holistic understanding of lipid hijacking by vector-borne diseases is on the horizon.

Collectively, we aimed to shed light on the unifying principle of lipid utilization in vector-borne diseases. Research in this field is essential for our understanding of mammalian pathogenesis and disease transmission by arthropods. We hope that continuing research in arthropod biology and understudied microbes will provide a greater understanding of host-vector-pathogen interactions.
